# 3D tumor explant as a novel platform to investigate therapeutic pathways and predictive biomarkers in cancer patients

**DOI:** 10.3389/fimmu.2022.1068091

**Published:** 2022-12-14

**Authors:** Monica Rodolfo, Veronica Huber, Mara Cossa, Gianfrancesco Gallino, Biagio E. Leone, Viviana Vallacchi, Licia Rivoltini, Elisabetta Vergani

**Affiliations:** ^1^ Department of Experimental Oncology, Translational Immunology Unit, Fondazione IRCCS Istituto Nazionale dei Tumori, Milan, Italy; ^2^ Department of Pathology, Fondazione IRCCS Istituto Nazionale dei Tumori, Milan, Italy; ^3^ Melanoma and Sarcoma Surgery Unit, Fondazione IRCCS Istituto Nazionale dei Tumori, Milan, Italy; ^4^ Department of Medicine and Surgery, University of Milano-Bicocca, Monza, Italy

**Keywords:** patient-derived 3D tumor explants, bioreactor, immunotherapy, melanoma, sarcoma

## Abstract

Immunotherapy with immune checkpoint inhibitors can induce durable clinical responses in different human malignancies but the number of responding patients remains globally modest. The limited therapeutic efficacy of ICI depends on multiple factors, among which the immune suppressive features of the tumor microenvironment play a key role. For this reason, experimental models that enable dissection of the immune-hostile tumor milieu components are required to unravel how to overcome resistance and obtain full-fledged anti-tumor immunity. Recent evidence supports the usefulness of 3D *ex vivo* systems in retaining features of tumor microenvironment to elucidate molecular and immunologic mechanisms of response and resistance to immune checkpoint blockade. In this perspective article we discuss the recent advances in patient-derived 3D tumor models and their potential in support of treatment decision making in clinical setting. We will also share our experience with dynamic bioreactor tumor explant culture of samples from melanoma and sarcoma patients as a reliable and promising platform to unravel immune responses to immune checkpoint inhibitors.

## Introduction

Rapid scientific and technological advance is revolutionizing treatment options and research tools in the era of personalized cancer therapy. The advent of immune checkpoint inhibitors (ICI) targeting the immune system has found wide application across cancer histotypes and represents the principal therapeutic intervention for advanced cancer patients (1). However, intrinsic and on-treatment resistance development remains a challenge of this therapeutic approach and major effort is dedicated to elucidate the involved mechanisms (2). In depth analysis of tumor biopsies by multi omics approaches has provided insight into the complex scenario prevailing at tumor site, but these approaches have a major limitation as they give only a snapshot of the actual conditions and ignore tumor evolution during treatment (3, 4). Co-clinical studies based on systems allowing the in-toto culture of tumor biopsies and the maintenance of the genomic and morphological characteristics of the original tumor may contribute filling this gap. In particular, *ex vivo* 3D culture platforms, which maintain most of the features of tumor immune microenvironment (TIME) (5) and capture drug-mediated dynamic modulations, can elucidate molecular and immune-related mechanisms underlying clinical response or resistance to therapy (6). This approach could help gathering a comprehensive mechanistic overview and define predictive biomarkers of clinical outcome in patients treated with ICI (7). Here, we provide an overview of the relevance and potential implications of the tumor explant culture system in studying ICI responses. In addition, we show the advantages of a 3D platform of dynamic culture of tumor explants in a Bioreactor for profiling molecular and immunologic mechanisms induced by ICI in human metastatic melanoma (MM) and soft tissue sarcoma (STS) samples.

## Patient-derived models for cancer drug testing

The contribution of newly developed targeted drugs and immunotherapies to improve patient survival has been limited by the lack of appropriate experimental models maintaining original tumor microenvironment (TME) architecture and composition. So far, patient-derived xenografts (PDX) have become the preferred tool for drug testing, to identify novel tumor markers and therapeutic targets and to translate findings aimed at optimizing treatment of cancer patients. PDX models generated from freshly resected tumor specimens or from tumor-derived organoids transplanted into immunodeficient mice preserve the tumor intrinsic heterogeneity and can recapitulate the interactions of cancer cells with the surrounding live environment, but not with the human immune system (8). Humanized mice models grafted with human immune cells have been generated to bypass this problem (9). Nonetheless, animal models are time consuming and have high costs, and thus may not represent the rational choice for real-time precision cancer therapy. Moreover, the advent of immune based-therapies requires models able to mimic native TIME. In this scenario 3D culture systems represent a valid alternative.

3D models are classified into spheroids, organoids and patient-derived tumor explants (PDE), based on the structural complexity. Spheroids are aggregates of cells obtained from cancer cell lines or tumor biopsies, self-assembling in an environment that prevents attachment to a flat surface. They are of low complexity in mirroring tumor organization, but they may retain their endogenous extracellular matrix and many metabolic similarities to the original tissue (10). Organoids are mini-organs reconstituted and embedded in an extracellular matrix reproducing many structural and functional aspects of the parental organ. In addition to the use of pluripotent stem cells, tissue-derived tumors from patients can also be established as organoids, namely patient-derived organoids (PDO) (11). PDO typically recapitulate features and genetic characteristics of the parental tumor, as observed in PDO from colorectal and gastroesophageal cancer patients (12). Of note, organoids and PDO can be expanded and cryopreserved, a fundamental feature especially for rare cancer types (13). However, these models are characterized by the inability of preserving the native features of TIME, and even the exogenous addition of selected immune cell populations as a co-culture is insufficient to reproduce the required complexity to evaluate the response to immunotherapy.

A further level in TME complexity in 3D models is represented by PDE, that consist in the *ex vivo* culture of freshly-resected human tumor fragments. PDE recapitulate tissue architecture, TME and preserve the human immune system components, thus allowing the evaluation of drug responses in a 3D context. PDE preserve tumor-specific genetic alterations, transcriptomic profiles and histopathology of individual patients, allowing personalized drug screening and the identification of drug resistance mechanisms (14). LeBlanc and coworkers reported that glioblastoma-derived PDE largely retain genetic and transcriptomic heterogeneity of parent tumors, enabling the dissection of glioblastoma heterogeneity evolution during disease progression and treatment response (15). In a recent study, the inhibition of NOTCH signaling pathway in PDO of BRAFV600E/K601Q MM enhanced the sensitivity to the MEK inhibitor cobimetinib, thereby supporting the contribution of PDO models to identify therapy resistance mechanisms (16).

PDE, endowed with the native TIME, represent ideal *ex vivo* models for immunotherapy studies. In fact, deciphering the dynamic interactions between tumor and immune cells provides insights into the mechanisms regulating sensitivity or resistance to immune-based therapies including ICI. A further PDE model consisting of patient-derived tumor tissues embedded in collagen and termed organotypic tumor spheroids (PDOTS) maintains immune cell composition when cultured in microfluidic chips, and anticipates clinical benefit of PD-1 blockade based on cyto/chemokine release (17, 18). The addition of anti-PD-1 antibody to air-liquid interface (ALI) culture of PDO obtained from different cancer types, including MM, activates the PD-1-expressing CD3^+^ T cell compartment and induces cytotoxicity. The bystander increase in distance between CD8^+^ effector T cells and regulatory T cells (Treg) contributes avoiding Treg-mediated suppression of resident CD8^+^ T cells (19). Similarly, ICI efficacy in restoring anti-tumor activity of γδ T cell-based immunotherapy has been demonstrated in melanoma PDO (20, 21). In this setting, the secretion of cyto/chemokines, along with T cell activation levels measured in a PDE model of patient-derived tumor fragments (PDTF) from different tumor types embedded into an artificial extracellular matrix, could predict clinical response to PD-1 blockade (22). Similar observations were recorded following PD-1 inhibition in explant cultures of head and neck cancer, gastric and gastroesophageal adenocarcinoma (23, 24). Importantly, the PDTF platform can be also applied to identify suitable neoadjuvant treatment strategies in patients with early-stage cancer, as recently described for anti-CTLA-4 plus anti-PD-1 combined with IL-2 in PDE of checkpoint inhibitor-resistant melanoma patients (25).

## 3D culture models for personalized melanoma and sarcoma therapies

MM is generally characterized by a strong immune infiltrate. Its characteristically very high tumor mutational burden (TMB) and consequent tumor neoantigen expression determines its high responsiveness to ICI. However, the majority of patients demonstrate resistance either by lack of an initial response or by resistance acquisition during treatment (26). Even if tumor PD-L1 expression and TMB have been found to correlate with clinical responses to ICI in melanoma, they cannot accurately predict patient outcome due to the complex mechanisms driving immune responses (27). Multi omics analysis of tumor biopsies have identified other biomarkers predicting response of MM patients to ICI (3, 28). However, tumor biopsy does not represent a suitable system to intercept early ICI-derived immune responses, as selected modifications of resident immune cell phenotype and function possibly occur in the tumor evolving during therapy. This limitation could be overcome by TIME preserving melanoma *ex vivo* 3D platforms. So far, melanoma 3D culture models such as spheroids and organoids, skin reconstructs and melanoma-on-chip models that incorporate TME elements represent helpful tools to screen therapeutic agents (29). Nonetheless, limited experience exists in utilizing melanoma organoids for drug screening and preclinical studies, despite organoids can be readily generated from melanoma biopsies and can be supplemented with autologous immune cells for evaluation of immunotherapeutic responses (30). On the other hand, melanoma spheroids can be useful for lead-compound testing, as shown by the evaluation of the spheroid morphometric parameters in the screening of novel compounds like photodynamic therapy (31). To overcome MAPK-mediated radioresistance, 3D BRAF- and NRAS-mutant melanoma cell spheroids have been exploited to evaluate the efficacy of combined targeted radionuclide therapy and MEK inhibitor (32). Despite the lack of pre-existing immune infiltration, human organotypic skin melanoma cultures can be also used for the investigation of early events that regulate tumor-immunological mechanisms. In fact, this system consists in a reconstructed TME that closely resembles tumor growth, allowing the study of host-malignant cell interactions within a multicellular tissue architecture (33). Finally, melanoma PDO cultures in microfluidic 3D devices or embedded in collagen extracellular matrix represent a valid model to evaluate the effect of ICI and to predict patient clinical response (17, 22, 25).

STS are rare tumors of mesenchymal origin and encompass more than 80 histologies. In the last years, clinical trials testing ICI in advanced STS showed limited clinical activity in an unselected population of patients, with anecdotal durable responses to PD-1 blockade observed in undifferentiated pleomorphic STS and dedifferentiated liposarcoma. These STS histologies are characterized by a complex altered karyotype and are thus supposedly more immunogenic, more infiltrated by immune cells and therefore more responsive to ICI compared to translocated STS (34, 35). However, STS dichotomization in simple (skSTS) and complex karyotypes (ckSTS) does not reflect a sharp demarcation of immunogenicity. Recent studies highlighted unexpected associations between genetic and/or epigenetic features of tumor and immune composition of the TME, revealing a great heterogeneity also within the same STS type (36). Tertiary lymphoid structures (TLS), present in 20% of STS, and high tumor immune infiltration have been recently described as potential biomarkers of response to anti-PD-1 therapy (37). However, ICI resistance can be observed also in TLS-positive STS, and TME analysis revealed an enrichment of Treg in resistant TLS-positive STS compared to the sensitive counterpart (38). This indicates that a fine and systematic TME dissection is required to identify STS determinants of ICI response and resistance. Growing evidence promotes the suitability of 3D cultures biology and therapeutic efficacy studies in both STS and bone sarcoma. In Ewing-Sarcoma, spheroids enabled the evaluation of chemotherapeutic and targeted drugs and the identification of antigens for immunotherapy (39). Voissiere et al. showed in 2D and 3D chondrosarcoma models that doxorubicin resistant spheroids were sensitive to TH-302, a pro-drug activated in hypoxia, and reported that the sensitivity increased with spheroid dimensions (40). Similarly, STS organoids obtained from surgical specimens generated knowledge about STS biology, while synovial sarcoma PDO studies revealed epigenetic dysregulations (41). Of note, PDO from epithelioid sarcomas could be also established by ALI culture method (42). To evaluate efficacy of Wee1 inhibitor MK-1775, alone and in combination with gemcitabine, PDE models of undifferentiated high-grade sarcoma, malignant peripheral nerve sheath tumors, pleomorphic spindle cell sarcoma and osteosarcoma were successfully applied (43). Recently, PDO derived from STS subtypes were generated by mixing tumor cell suspensions with extracellular matrix hydrogel and with autologous immune cells to perform chemotherapy and immunotherapy screening, thus highlighting the potential application of PDO also in STS (44).

## PDE as an optimal 3D platform to investigate ICI treatment efficacy in melanoma and sarcoma

PDE implementation in clinical practice is still hampered by several limitations. They include the relative short-term viability of the cultures, as evidenced for endometrial PDE that remained viable only for one day, and the lack of a functional vascular system, impacting oxygen and nutrient diffusion and waste removal (14). To circumvent these limitations, an innovative 3D dynamic culture system, based on the use of the microgravity technology provided by the Rotary Cell Culture System (RCCS) Bioreactor was developed, enabling long-term culture of tissue explants.

This device was first dedicated to 3D tissue engineering of cartilage and human collagen bone matrix formation (45, 46), demonstrating how the RCCS-based fluid dynamic culture conditions influence tissue growth and regeneration. Such conditions favor 3D assembly of single cells into aggregates, as shown in the case of astrocyte-like or neuronal-like models reproducing neuronal features (47, 48). RCCS Bioreactor culture was further validated for its ability to preserve the morphological and functional features in multiple myeloma tissue components. These studies showed that seven days treatment with Bortezomib of a peritoneal myeloma PDE retrieved from a responsive patient decreased tumor viability and release of the pro-angiogenic factors VEGF and angiopoietin-2. Conversely, no effect was observed in a myeloma PDE retrieved from a non-responding patient, suggesting that RCCS Bioreactor-based cultures may reflect the drug response observed *in vivo* (49). Moreover, the simulated microgravity in RCCS Bioreactor-based cultures could modify the ultrastructure of human breast cancer cells and was accompanied by increased apoptosis and inhibition of their migration ability (50). In addition, simulated microgravity influenced metabolic pathways, such as lipid metabolism and the Krebs cycle in human breast cancer cells, osteoblasts, oligodendrocytes and gastric cancer cells (51–54). Of note, metabolomic analysis of supernatants of Erdheim-Chester disease (ECD) biopsies cultured for 4 days in RCCS Bioreactor revealed that the immunometabolic changes of trained immunity occurring in ECD lesions *in vivo*, such as increased glycolysis and accumulation of fumarate and malate, can be dampened by MAPK pathway inhibition (55). A recent study in gastric cancer showed that PDE culture in RCCS bioreactor recapitulates the action of chemotherapy in patients by affecting cell survival and by weakening the action of drug resistance-associated genes (56).

3D dynamic cultures of PDE in the RCCS Bioreactor can be reproducibly applied to study the effects of exposure to drugs, resistance mechanisms and molecular signaling occurring during the interactions between tumor cells and immune infiltrate (57). The absence of an ALI interface and the hydrodynamic forces acting inside the RCCS Bioreactor culture vessels permit efficient gas inter-exchange, thereby optimizing oxygen and nutrient supply, catabolite removal and drug tissue distribution. While 3D cultures are typically short-term cultures and thus amenable only to short-term drug treatments, one fundamental asset of this approach is the reliable long-term maintenance of the tissue, which permits up to fourteen days culture of differentiated tissues like tumors (46–49). This allows to investigate the modulating activities of drugs acting on cancer and/or TME and TIME.

**Figure 1:**
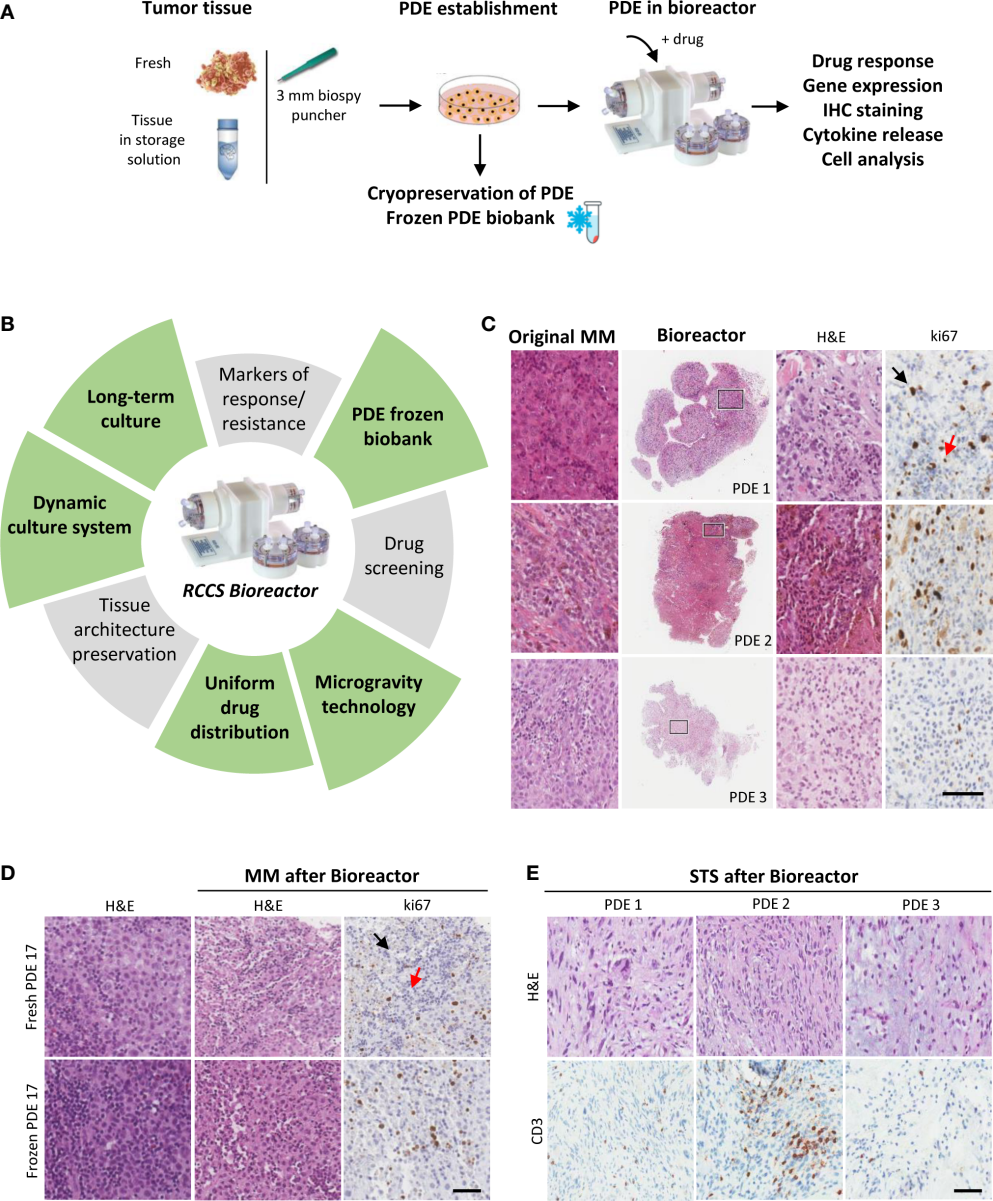
Dynamic 3D culture of PDE in RCCS Bioreactor preserves TIME architecture of MM and STS. **(A)** Strategy to establish tumor PDE to evaluate drug effects after culture in Bioreactor. **(B)** Main features common of PDE cultures (grey sectors) and those specific of PDE cultured in RCCS Bioreactor (green sectors). **(C)** Representative histological images of MM PDE after 3 days culture in Bioreactor compared to the original tumors. Right panels show higher magnification of the marked areas. **(D)** Representative images of ki67 stained fresh or frozen MM PDE before and after culture in Bioreactor. **(E)** Representative images of CD3 staining of frozen STS PDE after culture in Bioreactor. PDE 1: ckSTS, a myxofibrosarcoma of the extremities; PDE2: ckSTS, a retroperitoneal dedifferentiated liposarcoma; PDE 3: skSTS, a myxoid liposarcoma of the extremities. Ki67 staining of proliferating tumor cells and lymphocytes are indicated by black and red arrows, respectively. H&E, hematoxylin and eosin. Scale bar 80 μM **(C)**, and 200 μM **(D, E)**.

We set up a RCCS Bioreactor PDE platform at our unit to investigate mechanisms associated with response/resistance to ICI, with a particular focus on MM and STS. Samples obtained at surgical tumor resection were processed by a 3 mm biopsy puncher within 24 hours upon storage at 4°C in tissue storage solution to obtain PDE. PDE were cultured in RCCS Bioreactor upon drug treatment and then analyzed for drug response by gene expression profiling, tissue immunohistochemistry, cyto-chemokine release in culture supernatants (**Figure 1A**). PDE can be frozen in cell cryopreservation medium and banked in liquid nitrogen for prospective studies (Supplementary materials and methods). Several PDE can be obtained from one clinical sample, allowing comparative testing of different drugs and analysis of modifications at different time points. A particular feature of RCCS Bioreactor 3D cultures is the uniform tissue distribution of drugs, which is guaranteed by the low shear force in the rotating vessels (**Figure 1B**).

**Figure 2:**
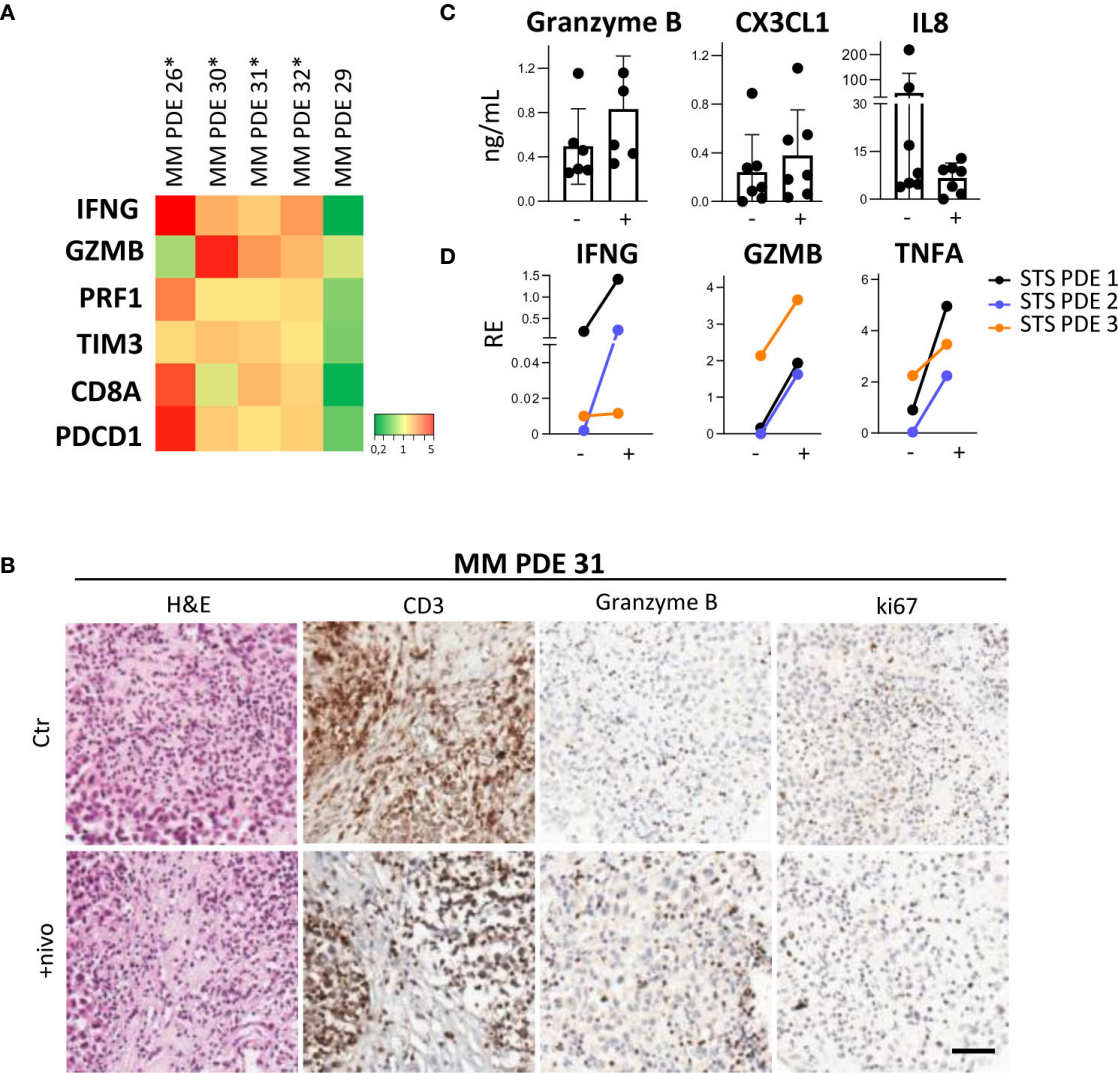
*Ex-vivo* treatment with nivolumab of 3D PDE in RCCS Bioreactor. **(A)** Heatmap showing gene expression levels in MM PDE deriving from lymph node metastases of treatment-naïve melanoma patients. Ratio of PDE treated with nivolumab and untreated control, p=0.013 by MANOVA. *: patients responding to adjuvant immunotherapy (absence of relapse at one year). **(B)** IHC staining of CD3, Granzyme B and ki67 in a representative case of MM PDE. Ctr: Control; +nivo: +nivolumab. **(C)** Granzyme B, CX3CL1 and IL8 in culture supernatants from MM PDE. **(D)** Expression of IFNG, Granzyme B (GZMB) and TNFA transcripts in PDE from STS after treatment compared to untreated control by qRT-PCR. p < 0.05 by paired Student’s t test. RE, Relative Expression. -, untreated control; +, nivolumab.

Our experiments show that melanoma PDE cultured for three days in RCCS Bioreactor vessels maintain the characteristics of the original tumors, including TME/TIME architecture and cellularity, viability of tumor cells and infiltrating lymphocytes, as indicated by ki67 staining of both cell types (**Figure 1C**). Moreover, this culture system prevented the efflux of infiltrating immune cells evidenced by the absence of CD45^+^ cells in culture supernatants (data not shown). A clear advantage of the RCCS Bioreactor PDE model system is the preservation of the original TME/TIME viability in banked tumor fragments frozen in vitality-preserving freezing buffer (**Figure 1D**). We could observe the preservation of tumor tissue histo-architecture and TME also in STS samples, as demonstrated in two representative cases of ckSTS, a myxofibrosarcoma of the extremities and a retroperitoneal dedifferentiated liposarcoma, and in one case of skSTS, a myxoid liposarcoma of the extremities (**Figure 1E**). These features of RCCS Bioreactor allow the creation of a biobank for simultaneous testing and retrospective evaluation. Exposure to BRAF/MEK inhibitors of PDE deriving from treatment naïve melanoma patients revealed determined a reduced number of ki67 proliferating melanoma cells (57). This setting also enabled us to evaluate T cell function after exposure to anti-PD-1 antibody of PDE obtained at baseline from patients clinically responding to adjuvant nivolumab, as demonstrated by the significant upregulation of a gene signature associated to immune response activation (IFNG, GZMB, PRF1, CD8, TIM3, PDCD1) (**Figure 2A**). PDE tissue immunostaining confirmed the increase of Granzyme B positive cells compared to untreated control samples (2-5 fold increase), together with a reduction of ki67 positive proliferating melanoma cells (**Figure 2B**). The analysis of PDE culture supernatants revealed also a boost in the release of Granzyme B and Fractalkine/CX3CL1 and the decrease of IL-8, confirming the immune modulation after PD-1 blocking (**Figure 2C**). Similar results were obtained in STS samples, where treatment with nivolumab increased the gene expression of the T cell activation markers IFNγ, Granzyme B and TNFα (**Figure 2D**). As in MM, also in STS, banking of tumor samples for 3D testing should be included in prospective studies to allow investigations of personalized therapies.

## Discussion and conclusions

In comparison to PDX models, PDE, by maintaining the phenotype and TME of the individual tumor, may provide a realistic environment to assess the functional *ex vivo* response to drugs. Several studies highlighted the usefulness of PDE-based drug screening and their predictive value or association with clinical responses, confirming the usefulness of this system to prospectively assess individual patient response (7, 13, 58).

Our findings indicate that 3D RCCS Bioreactor-based culture of PDE can be exploited as a preclinical approach to investigate melanoma and sarcoma clinical response to current targeted and immune therapies (57). Of note, in a subset of melanoma explants from nivolumab responders, we measured increased IFNγ and Granzyme B levels, suggesting the activation of infiltrating T cell effector functions. We observed analogous effects in PDE from STS, representative of both ckSTS and skSTS. Our data suggest that *ex vivo* ICI treatment of PDE simulates an active antitumor immune response, thereby providing a proof-of-concept strategy to identify patients who might benefit from such kind of treatment. Our results are in line with the numerous other investigators operating in the 3D cancer model field (15, 17, 20, 22, 24, 25) and we encourage the establishment in the clinics of personalized *ex vivo* platforms to test immunotherapy efficacy, define prediction markers and develop combination strategies. We underline that preclinical or co-clinical testing of immunotherapeutic agents must rely on 3D models, such as PDO (59), PDOTS (17) or PDTF (22), representing the most accurate patient-tailored TME replicas. By leveraging cryopreserved PDE, this system permits comparing retrospective samples from immunotherapy responders vs non-responders to unravel the local immunomodulatory effects and identify predictive markers. Based on our experience, cultures of frozen PDE retain the same morphology characteristics as cultures derived from fresh PDE regardless of freezing and storage time, supporting the creation of multicentric PDE biobanks. In a desirably close future, routine 3D personalized testing may improve accuracy and efficiency of personalized therapeutic approaches ultimately impacting clinical outcome of cancer patients.

## Data availability statement

The original contributions presented in the study are included in the article/**Supplementary Material**. Further inquiries can be directed to the corresponding author.

## Ethics statement

The study was approved by the Independent Ethic Committee and by the review board of Fondazione IRCCS Istituto Nazionale dei Tumori (protocol INT92/17 and INT85/10) and written informed consent was obtained from patients.

## Author contributions

MR and EV set up the system, supervised the experiments, wrote and revised the manuscript. MC, BEL, and GG selected and analyzed specimens. EV performed experiments. EV, VV, and MR analyzed the results. VH and LR wrote and revised the manuscript. All authors critically revised the manuscript. All authors contributed to the article and approved the submitted version.
