# Supplementary material for: 3D tumor explant as a novel platform to investigate therapeutic pathways and predictive biomarkers in cancer patients
Source: Front Immunol. 2022 Dec 14;13:1068091. doi: 10.3389/fimmu.2022.1068091 (PMC9794575; doi:10.3389/fimmu.2022.1068091)
Supplement: Supplementary file 1 [file Presentation_1.pdf]

Supplementary materials and methods

3D cultures of tumor explants in RCCS bioreactor

PDE fragments were obtained as cubes by a 3 mm biopsy puncher (Integra Miltex Princeton, NJ, USA) from fresh tumor specimens or from tissue maintained in MACS Tissue Storage Solution (Miltenyi Biotec, Bergisch Gladbach, Germany) for up to 24 hours. PDE fragments were tested fresh or were cryopreserved in cell freezing medium (RPMI 1640 + 30% FBS + 10% DMSO) for banking in liquid nitrogen for prospective studies. PDE were cultured in duplicate in the RCCS bioreactor chambers (RCCS Bioreactor, Synthecon, Houston, TX, USA) for 3 days, in culture medium and in the presence of nivolumab at 50 µg/mL (OPDIVO, Bristol Myers Squibb, NY, USA). When recovered, samples were halved, a half was fixed in formalin and paraffin-embedded for histopathology, and a half was fixed in RNAlater Stabilization Solution (ThermoFisher Scientific) for RNA analysis. Protein release in culture supernatants were tested by Cytometric Bead Array (BD Biosciences, Franklin Lakes, NJ, USA).

RNA extraction and qRT-PCR analysis

RNA was extracted from MM and STS PDE with the NucleoSpin RNA isolation kit (Macherey Nagel, Bethlehem, PA, USA), quantified using Qubit fluorometer and Qubit RNA HS assay kit (Q32852, ThermoFisher Scientific, Waltham, MA, USA), reverse-transcribed using the High-Capacity cDNA Kit, and pre-amplified using a Preamp Master Mix Kit according to the manufacturer’s instructions (ThermoFisher Scientific). qRT-PCR was carried out in triplicate and run on QuantStudio 7 Flex instrument and analysis was performed using SDS software QuantStudio 7 Flex software. The results were calculated with the $2^{-\Delta Ct}$ method using GUSB or RPL13A as housekeeping gene for MM and STS samples, respectively. TaqMan assays for IFNG (Hs00959291_m1), GZMB (Hs04261345_m1), PRF1 (Hs00169473_m1), TIM3 (Hs00958618_m1), CD8A (Hs00233520_m1), PDCD1 (Hs01550088_m1), TNFA (Hs00174128_m1), RPL13A (Hs03043885_g1) and GUSB (Hs00939627_m1) were used.

Immunohistochemistry

Immunohistochemistry was performed manually on PDE tissue sections by staining with specific antibodies against ki67 (M7240, Dako Agilent, Glostrup, Denmark), CD3 (A0452, Dako Agilent) and Granzyme B (NCL-L-GRAN-B, Leica Microsystems, Wetzlar, Germany) after antigen retrieval (0.5 mM EDTA pH 8, for 15 or 20 minutes), using a peroxidase labelled polymer (UltraVision Quanto Detection System HRP Polymer, Thermo Fisher Scientific), and brown DAB as a chromogen (Dako). Antibody incubation of PDE sections was performed overnight at 4°C as by manufacturer recommendation using ki67 antibody at 1:100 dilution, and CD3 antibody and Granzyme B antibody diluted 1:50. Staining in tonsil tissue was used as positive control, while staining without antibodies was used as negative control. Ki67 positive cells in PDE sections were quantified by the pathologist as the average number of cells per high-power field. Stained sections were scanned using the Aperio ScanScope XT systems (Aperio Technologies, Leica Microsystems).
